# Postpartum nurses' perceptions of barriers to screening for intimate partner violence: a cross-sectional survey

**DOI:** 10.1186/1472-6955-11-2

**Published:** 2012-02-20

**Authors:** Margaret E Guillery, Karen M Benzies, Cynthia Mannion, Sheila Evans

**Affiliations:** 1 Alberta Health Services, Rockyview General Hospital 7007 14th Street Calgary Alberta T2V 1P9, Canada; 2 Faculty of Nursing, University of Calgary, 2500 University Drive NW, Calgary, Alberta T2N 1N4, Canada

## Abstract

**Background:**

Intimate partner violence (IPV) is a human rights violation that is pervasive worldwide, and is particularly critical for women during the reproductive period. IPV includes physical, sexual and emotional abuse. Nurses on in-patient postpartum units are well-positioned to screen women for IPV, yet low screening rates suggest that barriers to screening exist. The purpose of this study was to (a) identify the frequency of screening for IPV, (b) the most important barriers to screening, (c) the relationship between the barriers to screening and the frequency of screening for types of abuse, and (d) to identify other factors that contribute to the frequency of screening for IPV.

**Methods:**

In 2008, we conducted a cross-sectional survey of 96 nurses from postpartum inpatient units in three Canadian urban hospitals. The survey included the Barriers to Abuse Assessment Tool (BAAT), adapted for postpartum nurses (PPN). Ordinary least squares (OLS) regression models were used to predict barriers to screening for each type of IPV.

**Results:**

The frequency of screening varied by the type of abuse with highest screening rates found for physical and emotional abuse. According to the BAAT-PPN, lack of knowledge was the most important barrier to screening. The BAAT-PPN total score was negatively correlated with screening for physical, sexual, and emotional abuse. Using OLS regression models and after controlling for demographic characteristics, the BAAT-PPN explained 14%, 12%, and 11% of the variance in screening for physical, sexual and emotional abuse, respectively. Fluency in the language of the patient was negatively correlated with screening for each type of abuse. When added as Step 3 to OLS regression models, language fluency was associated with an additional decrease in the likelihood of screening for physical (beta coefficient = -.38, *P *< .001), sexual (beta coefficient = -.24, *P *= .05), and emotional abuse (beta coefficient = -.48, *P *< .001) and increased the variance explained by the model to 25%, 17%, and 31%, respectively.

**Conclusions:**

Our findings support an inverse relationship between rates of screening for IPV and nurses' perceptions of barriers. Barriers to screening for IPV, particularly related to knowledge and language fluency, need to be addressed to increase rates of screening on postpartum units.

## Background

Intimate partner violence (IPV) is a human rights violation that is pervasive worldwide, and crosses all social, economic, racial, ethnic, and cultural boundaries [1]. In a Canadian sample estimated to represent 653,000 women, 7% reported IPV of such severity that they feared for their lives, suffered serious injuries, and sought medical assistance [2]. Women who experience IPV can suffer a variety of long term health consequences, including depression [3-5] anxiety [6], and physical harm [4,7-9]. In Canada, health care costs for IPV-related injuries are estimated to be $4.2CDN billion annually [2]. IPV that occurs during the reproductive period creates additional risks for the mother and foetus. In a representative Canadian sample (*N *= 6,421) of biological mothers 15 years and older who gave birth in 2006, 10.9% experienced IPV within the last 2 years, and 3.3% reported one or more occurrences during their pregnancy [5]. IPV-related injuries result in increased reproductive health risks including placental abruption [10], preterm labour [5,6,10-13], preterm birth [14], antepartum haemorrhage [15], delivery of a low birth weight infant [10,16], and chorioamnionitis [17]. Pregnant women incurring kicks or blows to the abdomen from their partner are sufficiently injured to be admitted to hospital [16,18]. However, not all women seek treatment when injured. In a US study of 3,542 women who experienced and reported IPV during the postpartum period, 77% were injured, but only 23% received medical treatment for their injuries [19]. Women who experience IPV are more likely to be identified during health care encounters if screening occurs [7,20,21].

### Barriers to screening for IPV in health care settings

Generally, screening for IPV is conducted in physician offices [22,23], clinics [22-24], outpatient areas [25], and emergency departments [20,22,26-29]. In these health care settings, reported barriers to screening for IPV included (a) lack of privacy to screen [26,30], (b) language barriers [15,29-32], (c) cultural barriers [31,32], (d) lack of knowledge about IPV [26,29], (e) lack of information about screening tools, personal perceptions and feelings about domestic violence, (f) lack of time to screen [26,29], (g) lack of instruction on how to ask questions about abuse [29], (h) a personal or family history of abuse [29], (i) not knowing what to do in the event of disclosures, and (j) fear of shocking the patient [32]. Nurses on inpatient postpartum units are well-positioned to screen women for IPV due to the one-to-one, intimate care provided. The trust relationship developed between nurses and postpartum women can be an important precursor to women's willingness to disclose IPV [33]. In spite of professional responsibility to screen for IPV [34,35], screening rates are low [28,36], suggesting that barriers exist that prevent nurses from screening for IPV. The aim of this study was to explore perceptions of barriers to screening for IPV on postpartum units in a sample of Canadian nurses.

### Risk factors associated with IPV during the reproductive period

IPV is cyclical and may be misunderstood or unrecognized by the victimized woman [37]. During the reproductive period, risk factors for IPV include minority status [38], young maternal age [38], single, separated or divorced marital status [38], unstable partner relationship [32], low education [38,39], and low income [39].

### Variation in rates of IPV during the reproductive period

Pregnancy may trigger higher rates of IPV for some women [40], while acting as a protective mechanism for others [41]. In a representative sample of Canadian women (N = 6,421), 47% of women who were exposed to IPV during pregnancy reported a decrease in abuse, 5.4% reported an increase while the remainder reported that amount of abuse stayed the same [5]. This finding is similar to others [10] who reported no increase in IPV during pregnancy. In other studies, IPV is reported to escalate during the postpartum period [19,42-44], and for up to 33 months post delivery [39].

Peaks of IPV in the reproductive period vary by the type of abuse (i.e., physical, sexual, and psychological) [41]. In a sample of low-income women, IPV victims (*n *= 31) and comparison participants (*n *= 45) reported that rates of physical abuse peaked for victims of IPV during the first 3 months of pregnancy and then declined [41]. Rates of physical abuse were lower in the comparison group than the IPV victim group, with the highest rates occurring 12 months before pregnancy and during the 7 to 12 months after infant delivery [41]. In both the victim and comparison groups, rates of sexual and emotional abuse were highest during the month following infant delivery [41]. In another study, physical abuse peaked during the first 6 months of pregnancy, while sexual and psychological abuse peaked in the month after delivery [45]. Thus, there is some indication that screening during the postpartum period may be an opportune time to identify current sexual and emotional abuse. However, emotional abuse frequently accompanies physical abuse [37,44] and any abuse during the perinatal period was found to be predictive of later abuse [41]. While there are inconsistencies in the literature about risk for IPV during pregnancy [38], it is clear that there is a substantially increased risk for IPV during portions of the reproductive period for some women.

Screening for IPV during the postpartum period may be a timely opportunity to prevent subsequent abuse and poor outcomes; yet screening rates are low [28,36]. This suggests that there are barriers that need to be addressed to enable nurses to conduct appropriate screening. The purpose of this study was to determine (a) the frequency of screening for IPV on postpartum units, (b) the most important barriers to screening for IPV as identified by postpartum nurses, (c) the relationship between the barriers to screening for IPV and the frequency of screening for types of abuse, and (d) and to identify other factors that contribute to the frequency of screening for IPV by PPNs.

## Methods

### Participants

A cross-sectional survey was conducted with nurses from the postpartum units in all three hospitals that provide maternity care for 17,500 births annually [46] in a large Canadian city. Nurses from any practice position (staff nurse, educator, and managers) were eligible to participate if they were employed (full-time, part-time, or casual) on one of the units in 2008. Of the 291 nurses eligible to participate, 96 returned a completed survey (33% response rate). To maintain nurses' anonymity, minimal socio-demographic data were collected. See Table 1. There were 76 Registered Nurses (RNs) and 20 Licensed Practical Nurses (LPNs) who participated; all were female. The average age of nurses in the study was 41.8 years (SD 11.64), which was similar to the average age of RNs (58% were > 41 years) [47] and LPNs (41.2 years) [48] practicing in the province of Alberta in 2008. Of the 76 RNs, half held a diploma and half held a baccalaureate degree, which is similar to the average educational levels of practicing RNs in the same year [47].

**Table 1:** Socio-demographic characteristics of nurses (N = 96)

	*M (SD)*	*n (%)*
Age (years)	41.8 (11.64)	
Years of Practice	17.4 (14.0)	
Marital Status		
Partnered		64 (68.1%)
Not partnered		28 (31.9%)

### Measures

The Barriers to Abuse Assessment Tool (BAAT) was adapted with permission from the author [49] for use with nurses on postpartum units (BAAT-PPN). Minor adaptations included changes to (a) the name of the nursing practice setting (i.e., postpartum versus labor and delivery), (b) nursing skill mix (i.e., addition of LPNs), and (c) titles for nursing practice licensing organizations. The BAAT-PPN consisted of 31 items grouped into six subscales: Systemic (nine items, e.g., *lack of hospital protocol for abuse assessment*), Ethical (two items; e.g., *should not assess for abuse if necessary support and resources are lacking*), Knowledge (four items; e.g., *inadequate knowledge about the phenomenon of pregnancy abuse*), Personal (six items; e.g., *pregnancy abuse is a private problem*), Fear (four items; e.g., *retaliation by the partner that is directed at me*), and Nursing Role Barriers (six items; e.g., *the issue should be left to the experts and is not the domain of nursing practice*). Each item was rated on a 4-point Likert scale ranging from 1 (*strongly disagree*) to 4 (*strongly agree*). The theoretical range of scores was 31 to 124, with higher scores indicating greater barriers to screening for IPV. Items for each subscale were summed to create a subscale score; subscale scores were summed to create a total score. For this study, the internal consistency reliability (Cronbach's alpha) for the BAAT-PPN total score was .81. Cronbach's alphas for the subscale scores ranged from .50 (Ethical Barriers) to .83 (Systemic Barriers). The Spearman-Brown Prophecy stating that Cronbach's alpha increases with increasing number of items on the scale may explain the low alpha (.50) for the Ethical Barriers subscale that contained only two items [50].

The BAAT-PPN was pilot tested with 10 PPNs from a variety of practice positions (e.g., staff nurses, patient care managers, and nurse educators). During the pilot test, language fluency was identified as a barrier to screening for IPV in a multicultural context, and was added as a separate item to the survey. The item, *I do not assess a woman for abuse if she does not speak and understand a language in which I am fluent*, was rated on a 4-point Likert scale ranging from 1 (*strongly disagree*) to 4 (*strongly agree*).

The frequency of screening was captured by three items related to each type of abuse (e.g., *Please indicate how often you assess your patients for physical abuse)*. Respondents rated the frequency of screening for each type of abuse on a 4-point Likert scale ranging from 1 (*never*) to 4 (*always*). To identify the single most important barrier to screening, respondents were asked to check one BAAT-PPN subscale from a list. Additional factors potentially related to frequency of screening, such as participants' previous and current experiences with professional abuse and IPV were captured by six items. For example, the item, *Are you presently in an abusive relationship with an intimate partner/spouse*, was rated as *no *or *yes*. Socio-demographic variables were captured using items designed by the investigators for this study and included post-secondary education, years of practice, birth date, and marital status.

### Procedure

Ethical and administrative approvals were obtained from the Conjoint Health Research Ethics Board (ID #21471). Following information sessions, PPNs were invited to complete the survey. The unit clerk placed an envelope containing an information sheet about the study and an anonymous survey in each PPN's mailbox on the postpartum unit. Consent to participate was implied by return of a completed survey to a locked drop-box near the nursing station. Surveys were collected several times per week between March and May 2008. Participants who returned a survey were eligible to enter a draw for three $50CAD gift certificates to a family restaurant.

### Data analyses

Data were examined for outliers; two outliers were retained because of their potential clinical significance. Nurse age was missing for 19% of cases. There were no significant differences on scores for the dependent variables nor or any other socio-demographic variables between nurses who reported their age and those who did not. Missing values were replaced with the mean age for the sample. Data were examined for normality using histograms, stem and leaf plots, and Q-Q plots. On BAAT-PNN, except for the score on the Ethical Barriers subscale that was positively skewed, all other subscale and total scores approximated a normal distribution. Means/standard deviations and frequencies/percentages were calculated to describe the sample and scores on the BAAT-PPN and frequency of screening for types of abuse. Scores on the frequency of screening for types of abuse were treated as continuous variables because the categories were ordered and mutually exclusive [51]. Except for the frequency of screening score for sexual abuse that was positively skewed, all other frequency of screening scores approximated a normal distribution. There were no significant differences between RNs and LPNs in their frequency of screening for any type of IPV, *F*(1,94) = .80, *p *= .49; RN and LPN data were combined for analyses. PPN age and years of practice were significantly correlated with frequency of screening for physical and sexual abuse, and employed as covariates in regression analyses. No PPN characteristics were correlated with frequency of screening for emotional abuse. The relationships between additional factors and the frequency of screening were explored using Pearson's correlations. Controlling for covariates, three hierarchical ordinary least squares (OLS) regression models were calculated to identify the relationships between barriers and the frequency of screening for physical, sexual, and emotional abuse using the BAAT-PPN. In Step 1, demographic covariates were inserted when indicated. In Step 2, the BAAT-PPN total score was added. Data were analyzed using the Statistical Package for the Social Sciences (SPSS) version 19.0. The level of significance was set at *p *< .05.

## Results

### Frequency of screening for IPV

A minority of PPNs reported that they *never *screened for physical (7.3%), sexual (45.8%), or emotional (7.3%) abuse. The majority screened *sometimes *for physical (59.4%), sexual (42.7%), or emotional (56.3%) abuse. The remainder of PPN's reported that they screened *always *or *often *for physical (33.4%), sexual (11.5%), and emotional (36.5%) abuse.

### Barriers to screening for IPV

The means and standard deviations for the BAAT-PPN subscales and total score are presented in Table 2. PPNs were asked to identify the most important barrier to screening for IPV. Over one-third (37.6%) reported that Knowledge was the most important barrier. The second most important barrier was Systemic (29.0%), followed by Ethical (10.8%) and Nursing Role (10.8%). Few PPNs reported that Personal (5.4%) and Fear (6.5%) barriers were the most important barriers to screening for IPV on a postpartum unit.

**Table 2:** Number of items, range of scores, and means and standard deviations for BAAT-PPN subscale and total scores (N = 96)

BAAT-PPN	*Number of Items*	*Range of Scores*	*M (SD)*
Systemic Barriers	9	14-36	25.54 (4.50)
Ethical Barriers ^a^	2	2-8	4.21 (1.25)
Knowledge Barriers	4	5-16	10.53 (2.02)
Fear Barriers	4	5-16	10.53 (2.02)
Personal Barriers	6	2-15	10.18 (2.71)
Nursing Role Barriers ^a^	6	4-23	12.49 (3.15)
Total Score	31	32-95	73.32 (9.76)

*Note*. ^a ^*N *= 95 due to missing value. BAAT-PPN = Barriers to Abuse Assessment Tool-Postpartum Nurses

### Barriers to screening and frequency of screening for type of abuse

The BAAT-PPN Total score was negatively correlated with screening for physical, sexual, and emotional abuse (see Table 3). Ethical and Personal barriers on the BAAT-PPN were not correlated with the frequency of screening for any type of abuse. Systemic barriers, such as lack of hospital protocols and screening tools, were related to the frequency of screening for physical and emotional, but not sexual abuse. Knowledge and Fear barriers were related to the frequency of screening for each type of abuse. Nursing Role barriers were related to the frequency of screening for emotional abuse, only.

**Table 3:** Pearson's correlation between barriers to screening for IPV and frequency of screening for type of abuse (N = 96)

PPN Characteristic	Physical Abuse	Sexual Abuse	Emotional Abuse
Systemic	-.269*	-.183	-.222*
Ethical	-.076	.005	-.093
Knowledge	-.271*	-.260**	-.287**
Fear	-.271*	-.260**	-.287**
Personal	-.063	-.056	-.147
Nursing Role	-.187	-.198	-.221*
Total BAAT-PPN	-.328**	-.275**	-.351***

*Note*. BAAT-PPN = Barriers to Assessment Tool-Postpartum Nurses

* *p *< .05. ** *p *< .01. *** *p *< .001

### Relationships between barriers and frequency of screening

Controlling for PPN socio-demographic covariates, the variance (Adjusted R^2^) in PPN screening for physical, sexual, and emotional abuse explained by the BAAT-PPN was 14%, 12%, and 11%, respectively. As expected in each model, there was a negative relationship between barriers and the frequency of screening for each type of abuse. That is, the greater the barriers to screening, the lower the frequency of screening.

### Relationship between language and frequency of screening

In testing for additional factors, only the nurse's fluency in the patient's language was correlated with screening for physical (*r *= -.43), sexual (*r *= -.32), and emotional (*r *= -.55) abuse. To explore this variable as a barrier to the frequency of screening for types of abuse, three additional hierarchical OLS regression models were calculated with language fluency added as Step 3 (see Table 4). With the addition of the language fluency item, the variance explained in screening for physical, sexual, and emotional abuse by the BAAT-PPN increased to 25%, 17%, and 31%, respectively.

**Table 4:** Hierarchical ordinary least squares regression models for variables predicting the frequency of screening for types of abuse using the BAAT-PPN and language barrier (N = 96)

	Frequency of Screening for Types of Abuse					
	**Physical^a^**		**Sexual^b^**		**Emotional**	

**Predictor variable**	***ΔR^2^***	**β**	***ΔR^2^***	**β**	***ΔR^2^***	**β**
Step 1						
Control variables	.04		.06		-	-
**Step 2**						
BAAT-PPN	.10	-.33***	...06	-.28**	.13	-.37***
**Step 3**						
Language Barrier	.15	-.38***	.11	-.24*	.18	-.48***
Total Adjusted *R^2^*	.25***		.17**		.31***	

*Note*. BAAT-PPN = Barriers to Abuse Assessment Tool - Postpartum Nurse

^a ^Control variable = nurse age

b Control variables = nurse age and years of practice

*p < .05. ** *p *< .01. *** *p *< .001

## Discussion

This study is among the first to report screening practices and barriers to screening for IPV by Canadian nurses on post-partum units. In this study, the majority of PPNs reported screening their patients for physical or emotional abuse at least some of the time. Screening rates for sexual abuse were much lower; nearly half of PPNs never screened for this type of abuse. Rates of screening for IPV vary across contexts and health care professionals [32,52-55]. In a US study of emergency department medical records, [52] it was reported that only 9% of health care professionals documented screening for IPV. However, this report conflicted with registered nurses' self-reported rates (45%) of screening in the same emergency department. Others have suggested that 12% of all adult patients in the emergency department were screened for IPV [53]. In a chart review to assess clinician screening practices for domestic violence in a US Emergency Department, only 29% of women (*N *= 527) aged 18 to 65 had ever been screened [55]. Of those women who were screened using a single item question embedded in the medical history, 15% were identified as being victims of IPV. In population-based telephone survey, only 7% of women reported ever having been screened for IPV [54]. In a European study involving 56 healthcare practitioners (social workers, gynaecologists and midwives) in an obstetrical clinic, the researchers found that 4 health care providers routinely screened pregnant women for IPV, while the remaining 52 screened only if they suspected abuse given physical evidence or recurrent complaints of physical or somatic symptoms by the patient. Although the rates of screening for IPV appear to be higher in this study than others, there is no documented evidence to corroborate this self-reported rate of screening. A unique contribution of this study is that rates of screening for IPV may vary by the type of abuse: physical sexual, or emotional.

### Barriers to screening for IPV

Findings from this study suggest that systemic barriers contributed to low rates of screening for IPV. PPNs indicated that they did not have an appropriate hospital protocol or forms for documentation. PPN's reported a lack of privacy and time to develop trusting relationships as a barrier to screening, which is consistent with other reports [15,29,30,56-61]. For example, a study of barriers to screening for IPV in nurses from 10 US sites reported that 91.7% of maternity nurses (*N *= 385) ranked lack of privacy as the number one barrier [58]. Time constraints and being unsure of actions to take in the event of disclosure were ranked second by 50% of respondents.

Similar to findings in this study, lack of knowledge was a barrier to screening for IPV in other studies [26,29,32,52,53,56,58,62]. For example, lack of knowledge prevented nurses from completing the implementation of a screening program for domestic violence [26]. These researchers reported that knowledge barriers existed, and needed to be addressed prior to any implementation of a screening program. Lack of knowledge of IPV may be partially attributable to deficiencies in content offered in nursing school curricula. Haagbloom, Hallberg, and Moller [63] found that very few nurses received any kind of formal education about IPV in their educational programs. Others recommended that nurses are well-positioned to take a leadership role to ensure the inclusion of this content in undergraduate curricula [26,64].

In this study, few PPNs reported that Personal and Fear barriers prevented them from screening for IPV on postpartum units. However, these PPNs were reluctant to mislabel the situation and some expressed fear of reprisal from patients' partners, also similar to other findings [15,61,65,66].

While the majority of PPNs believed that there may be more appropriate times to screen for IPV (i.e., prenatally or in the community), they also believed that screening for IPV was within the scope of their nursing practice. However, there appear to be inconsistencies in PPN beliefs about screening for IPV and subsequent screening practices. Given that PPNs do not routinely screen for IPV appears to contradict their reported belief that screening for IPV is within the scope of their nursing practice. These findings concur with the results of a US study of 1,265 occupational health RNs and LPNs [62]. Both nursing groups considered screening for domestic violence was part of their nursing role, yet 71% were unaware or unsure of the existence of a written IPV policy in their workplace.

### Relationship between barriers to screening for IPV and frequency of screening

A unique finding in this study was that barriers to screening for IPV varied by type of abuse. Systemic barriers were negatively related with frequency of screening for physical and emotional, but not sexual abuse. Knowledge and Fear barriers were negatively related to the frequency of screening for each type of abuse. Nursing Role barriers were negatively related to the frequency of screening for emotional abuse only. Future research will be required to explore this unique finding. Programs supporting nurses' screening for IPV should consider that there may be different barriers related to screening for each type of abuse.

### Other factors related to the frequency of screening for IPV

In this study, lack of fluency in the patient's language affected PPNs frequency of screening for all types of abuse. Language was a stronger predictor of the frequency of screening than any PPN characteristics and the BAAT-PPN barriers. This finding is consistent with those of other researchers who have reported that nurses' lack of fluency in the language of their patients was a barrier to screening for abuse [15,30,58].

Nearly two-thirds of PPNs reported knowing a close friend or family member who experienced IPV. In the current study however, there were no significant relationships between professional or personal experiences with abuse and the frequency of screening for any type of abuse. In contrast, other researchers have found that a personal experience with abuse increased rates of reporting IPV [29,61]. Contrasting results may be related to the measurement tool or variations in practice settings.

This study was limited by a small sample of convenience, and a modest response rate, which limits the generalizability of the results to the population of PPNs. Given that the surveys were returned anonymously, a comparison between responders and non-responders could not be undertaken. Future studies with larger samples, and verification of screening practices using chart audits should be considered. The BAAT-PPN has limited psychometric evaluation. While it shows promise as a tool to assess barriers to screening for IPV during the reproductive period, it may be strengthened by the addition of a language fluency subscale.

## Conclusions

Inability to overcome the barriers to screening means a loss of opportunity to intervene and to break the cycle of IPV. IPV can be repeated throughout generations [67,68]. The barrier between fluency in the language of the patient and screening for IPV affects PPN nurses' decisions to screen. Along with nursing staff, hospital translator services and immigrant serving agencies should be involved in any discussions or plans to address the language fluency issue. Other strategies to overcome language barriers include the use of pictures, and other materials depicting IPV in various languages.

According to professional nursing associations, routine screening for IPV is considered a standard of competency for any nursing assessment [34,35]. Despite the expectations, PPNs in this study reported that they did not routinely screen for IPV. Therefore, there is a contradiction between expectations identified in nursing practice standards and actual nursing practice. Continuing education is required to address the lack of knowing how and when to assess for IPV and how to respond in the event of disclosure [64]. Nurse managers may wish to consider educational opportunities that include awareness of IPV policies, workshops, in-service training, and clinical practice time to rehearse screening procedures [26,57,69].

There have been numerous studies conducted of educational interventions to promote screening [30,70-73]. However, in these studies, barriers to screening for IPV were not identified until after the initial intervention had been implemented and low frequency of screening remained. According to Davis and Harsh [26], barriers to screening for IPV must be explored prior to implementation of a program to promote universal screening. This finding suggests that the presence of a screening tool and protocols are insufficient to ensure screening. Barriers affecting screening practices must first be identified and then addressed prior to implementing new screening policies and procedures.

## Competing interests

The authors declare that they have no competing interests.

## Authors' contributions

MG, KB, CM and SE designed the study. MG, KB, and CM analyzed the data and led the writing of the manuscript. All authors read and approved the final version.

## Pre-publication history

The pre-publication history for this paper can be accessed here:

http://www.biomedcentral.com/1472-6955/11/2/prepub
